# The KT Jeang retrovirology prize 2023: Thumbi Ndung’u

**DOI:** 10.1186/s12977-023-00632-9

**Published:** 2023-10-18

**Authors:** Thumbi Ndung’u

**Affiliations:** 1 Africa Health Research Institute, Durban, South Africa; 2 HIV Pathogenesis Programme, The Doris Duke Medical Research Institute, University of KwaZulu-Natal, Durban, South Africa; 3 Ragon Institute of MGH, MIT and Harvard University, Cambridge, MA USA; 4 Division of Infection and Immunity, University College London, London, UK

Thumbi Ndung’u grew up on a coffee farm in rural Kenya, where he received his primary and high school education at Gathugu Primary School and Nyeri High School. He studied Veterinary Medicine at the University of Nairobi and graduated in 1991, later joining the laboratory led by George Kinoti and Jasper Mumo at the same institute, to work on *Schistosoma haematobium* vaccine research. The project provided an opportunity for Ndung’u to travel to the laboratory of Donald Harn at the Harvard School of Public Health to train on hybridoma technology for making monoclonal antibodies. Besides the cultural shock of working overseas for the first time, this exciting six-month stint at Harvard was an eye-opener to cutting-edge biomedical research. Ndung’u subsequently returned to Nairobi where he worked to set up the infrastructure to produce monoclonal antibodies to *Schistosoma haematobium* egg antigens.

Ndung’u was elated when he was admitted with a full scholarship to pursue doctoral research at Harvard in 1995. He joined the laboratory of Max Essex, who was focusing on HIV genetic diversity and its implications for disease pathogenesis and the development of biomedical interventions against the virus. The decision to focus on HIV was driven by the emerging reality that HIV was rapidly spreading in Africa, and with no effective drugs or a vaccine at the time, it was obvious that this was going to be an important scientific challenge for Africa and the world. HIV/AIDS scientific discoveries were also rapidly emerging at the time, such as the design of highly effective antiretroviral drugs, understanding of the dynamics of HIV-1 replication in vivo, and the identification of HIV entry co-receptors and their genetic variations in human populations [1–6]. For his doctoral thesis research, Ndung’u generated the first infectious molecular clone of HIV-1 subtype C from a primary isolate of HIV-1. He also generated the first HIV-1 subtype C based simian human immunodeficiency virus chimera able to replicate in rhesus macaque cells [7–9]. Ndung’u received the Edgar Haber Award for outstanding thesis research. He also participated in other research in the laboratory to understand HIV-1 genetic variation and to study immune responses in non-B HIV-1 subtypes for vaccine development [10–13]. His work is important because subtype C HIV-1 is the most prevalent subtype globally and is predominant in southern Africa. In contrast, subtype B is more widely studied and is prevalent in Europe and the Americas.

Upon completion of his PhD, Ndung’u briefly worked under the mentorship of David Knipe as a postdoctoral fellow at Harvard, but soon decided to take a position to help lead efforts at a newly established HIV Reference Laboratory at the Botswana Harvard Partnership (BHP), a collaboration between the Botswana government and Harvard T.H. Chan School of Public Health, located in Gaborone, Botswana. The experience in Boston had been exhilarating, challenging and unforgettable, with the birth of his two children while both he and his wife were graduate students, and doing odd jobs to make ends meet while gaining superb training and savoring wonderful cultural experiences in the vibrant academic ecosystem and rich cultural history of New England. However, he was eager to play a direct role in research and the public health response to the now burgeoning epidemic in southern Africa.

The laboratory at BHP supported the national HIV/AIDS response and facilitated clinical trials of antiretroviral drugs and vaccine candidates. Ndung’u was involved in basic and translational laboratory research that complemented the ongoing clinical trials [14–18], helped to establish collaborative links with the University of Botswana and other institutions in the region, and co-supervised students. Although the clinical trials of antiretroviral drugs were highly successful in Botswana and elsewhere in Africa, setting the stage for the widespread roll-out of these drugs globally, the HIV-1 vaccine trials were unsuccessful. This led to the conclusion that development of an effective HIV-1 vaccine would require better understanding of HIV biology and protective immune responses [19]. Ndung’u then decided that his skills would be better utilized within a university setting, where he would also have an opportunity to interact with and train large numbers of students. In 2005, he moved to the University of KwaZulu-Natal in Durban, South Africa, to establish his own independent research group.

The University of KwaZulu-Natal provided a unique challenge due to the high burden of infection in the province of KwaZulu-Natal. However, there was an opportunity for ground-breaking work because of a committed and activist scientific community, superb research infrastructure, and community willingness to contribute to research. Supported by colleagues such as Hoosen (Jerry) Coovadia and Salim Abdool Karim, he developed a program of work on HIV-1 pathogenesis, anchored on acute HIV-1 infection studies and the heterogenous clinical outcomes that characterize HIV infection. With his students, he performed research on the role of immune factors, host restriction and HIV-1 replication cofactors on HIV-1 disease progression [20–25]. These studies demonstrated that regulation of expression and genetic variation of these factors could play a role in HIV-1 disease pathogenesis. Ndung’u also used cohort-based studies to explore the mechanisms of HIV-1 immune control that could be exploited for vaccine development. Several studies from his laboratory demonstrated that HIV-1 replication capacity was an important determinant of disease progression and that some “protective” human leukocyte antigen class I (HLA-I) alleles, associated with slow disease progression, mediated this effect [26–28]. These studies also defined the fitness landscape of HIV-1 subtype C Gag and Nef proteins, which are important targets for HIV vaccine design [29–31]. This work was performed in collaboration with the laboratories of Mark Brockman, Arup Chakraborty, and Bruce Walker. Other work in his laboratory showed that in individuals with protective HLA-I alleles, who naturally controlled subtype C HIV-1 infection, viral control was associated with broad CD8 + T cells that could efficiently kill the virus in ex vivo assays [32]. The Ndung’u group also showed that broad targeting of the HIV-1 Gag protein during acute HIV-1 infection is associated with relative control of the viral load set point but that these responses are not broad and rarely drive immune escape following infection, suggesting that they are defective [33–35]. Subsequent studies using HIV tetramers demonstrated that CD8 + T cell immune responses are broad during acute HIV-1 infection but are defective [36]. Taken together, these studies identified vulnerable regions of the virus that may be good candidates for a vaccine designed to induce effective T cell immune responses; in the case of immune escape they may still be effective due to debilitating mutations in the virus.

Ndung’u has also contributed to research on HIV transmission and spread. The HIV-1 epidemic is composed of diverse subtypes that differ in prevalence and rates of disease progression. His group found that HIV-1 subtype C has lower replication capacity (RC) than the less prevalent subtype B [37]. Moreover, in East Africa where multiple subtypes co-exist, there is a hierarchy in virus RC such that subtypes A and C have lower RC than D, which in turn has lower RC than inter-subtype recombinants. Subtype specific differences also extend to functions of specific viral genes known to play diverse roles in disease pathogenesis [38–40]. These differences may account for the reported differences in disease progression between subtypes and the differential spread of HIV-1 subtypes globally. Interestingly, in mother to child HIV-1 transmission, the group has shown that viruses with lower RC are preferentially transmitted [41].

In addition to research on HIV immunopathogenesis to inform vaccine development, the Ndung’u group also now studies HIV reservoirs and cure strategies. Most of this work is anchored on acute HIV-1 infection and natural controller cohorts. One cohort, known as the Females Rising through Support Education and Health (FRESH) has pioneered a unique approach that combines basic science research and social economic empowerment by providing intensive HIV-1 prevention services, career and life-skills training to address issues such as high school drop-out rates, gender-based violence and economic challenges facing young women in a South African Township [42, 43]. The study is investigating factors associated with HIV-1 acquisition and the virological and immunological impact of early antiretroviral treatment with the goal of informing vaccine and cure strategies [44–48]. The group is conducting a clinical trial to test whether a combination of broadly neutralizing antibodies and a toll-like receptor agonist can lead to HIV remission off antiretroviral therapy in women who initiated treatment during the acute phase of infection.

Ndung’u is currently the director for basic and translational science at the Africa Health Research Institute (AHRI) in Durban, South Africa and the scientific director of the University of KwaZulu-Natal’s HIV Pathogenesis Programme. He is a recipient of the South African Medical Research Council Gold Award and is a member of the Academy of Science of South Africa and an African Academy of Sciences Fellow. He is co-chair of the International AIDS Society Toward a Cure Board. Ndung’u is also this year’s recipient of the Harvard T.H. Chan School of Public Health Leadership Award in Public Health Practice. Ndung’u has trained more than 50 students and postdoctoral fellows, many of whom are now in leading roles in academia and other industries, and is passionate about contributing to training the next generation of scientific leaders on the African continent. A role he is therefore particularly excited about is that of programme director of the Sub-Saharan African Network for TB/HIV Research Excellence (SANTHE), an African-led research and capacity building consortium working in 8 African countries (South Africa, Cameroon, Uganda, Zimbabwe, Rwanda, Zambia, Kenya and Botswana).
